# Real-world characteristics and use patterns of patients treated with vericiguat: A nationwide longitudinal cohort study in Germany

**DOI:** 10.1007/s00228-024-03654-0

**Published:** 2024-03-12

**Authors:** Fabian Kerwagen, Christoph Ohlmeier, Thomas Evers, Stefan Herrmann, Inga Bayh, Alexander Michel, Silvia Kruppert, Joanna Wilfer, Rolf Wachter, Michael Böhm, Stefan Störk

**Affiliations:** 1 Department of Clinical Research and Epidemiology, Comprehensive Heart Failure Center, University Hospital Würzburg, D-97080 Würzburg, Germany; 2 Department of Internal Medicine I, University Hospital Würzburg, D-97080 Würzburg, Germany; 3 Bayer AG, Berlin, Germany; 4 Bayer AG, Wuppertal, Germany; 5 Bayer Vital, Leverkusen, Germany; 6 Bayer Consumer Care AG, Basel, Switzerland; 7 IQVIA Commercial GmbH & Co. OHG, Frankfurt am Main, Germany; 8 Department of Cardiology, University Hospital Leipzig, Leipzig, Germany; 9 Department of Internal Medicine Clinic III, Saarland University Hospital, Homburg/Saar, Germany

**Keywords:** Heart failure, Worsening heart failure, Vericiguat, Real-world, Pharmacoepidemiology

## Abstract

**Purpose:**

Vericiguat reduced clinical endpoints in patients experiencing worsening heart failure in clinical trials, but its implementation outside trials is unclear.

**Methods:**

This retrospective analysis of longitudinally collected data was based on the IQVIA™ LRx database, which includes ~ 80% of the prescriptions of the 73 million people covered by the German statutory health insurance.

**Results:**

Between September 2021 and December 2022, vericiguat was initiated in 2916 adult patients. Their mean age was 73 ± 13 years and 28% were women. While approximately 70% were uptitrated beyond 2.5 mg, only 36% reached 10 mg. Median time to up-titration from 2.5 mg to 5 mg was 17 (quartiles: 11–33) days, and from 2.5 to 10 mg 37 (25–64) days, respectively. In 87% of the patients, adherence to vericiguat was high as indicated by a medication possession ratio of  ≥ 80%, and 67% of the patients persistently used vericiguat during the first year. Women and older patients reached the maximal dose of 10 mg vericiguat less often and received other substance classes of guideline-recommended therapy (GDMT) less frequently. The proportion of patients receiving four pillars of GDMT increased from 29% before vericiguat initiation to 44% afterwards.

**Conclusion:**

In a real-world setting, despite higher age than in clinical trials, adherence and persistence of vericiguat appeared satisfactory across age categories. Initiation of vericiguat was associated with intensification of concomitant GDMT. Nevertheless, barriers to vericiguat up-titration and implementation of other GDMT, applying in particular to women and elderly patients, need to be investigated further.

**Supplementary Information:**

The online version contains supplementary material available at 10.1007/s00228-024-03654-0.

## Introduction

Heart failure (HF) is the most common cause of hospitalization in Germany [1]. One of the clinical hallmarks of HF is the occurrence of repeated episodes of worsening symptoms and signs, i.e. so-called worsening HF (WHF) events. WHF is considered an indicator of disease progression, and each episode of WHF increases the risk of further HF hospitalization or death [2].

There are multiple strategies for the prevention of episodes of WHF including pharmacological [3], and non-pharmacological treatments [4, 5]. In patients with HF and reduced ejection fraction (HFrEF), the current guidelines of the European Society of Cardiology (ESC) advocate drug treatment using all of the four foundational substance classes [6, 7]. In addition, for patients with a history of WHF, the soluble guanylate cyclase (sGC) stimulator vericiguat demonstrated incremental clinical benefit by reducing the risk of cardiovascular mortality or HF hospitalization in the VICTORIA trial [8]. Consequently, vericiguat is recommended by the ESC guidelines in symptomatic patients with chronic HFrEF and a history of WHF on top of the four foundational pillars [6]. Recently, the recommendation to initiate vericiguat in this high-risk HF population as early as possible to prevent further WHF events has been reaffirmed by the consensus statement of the Heart Failure Association of the ESC [2].

Despite these recent advances in pharmacotherapy, the implementation of guideline-directed medical therapy (GDMT) in clinical practice remains challenging [9]. For instance, the COVID-19 pandemic temporarily compromised the initiation of novel GDMT in Germany [10]. At the patient level, non-adherence to cardiovascular pharmacotherapy remains a major barrier in the care process [11–15]. Approximately only 50% of the patients with cardiovascular disease are consistently taking their prescribed medications, which has been suggested to contribute to up to 125,000 preventable deaths per year [12].

The assessment of reliable information on the implementation of GDMT is a prerequisite to understand potential drivers and barriers, and finally to improve clinical practice. So far, no study has investigated the real-world characteristics and drug use patterns of patients treated with vericiguat since its availability in September 2021 [16].

## Methods

### Study design and database

For the current retrospective analysis of longitudinally collected routine data, we used the anonymized claims data of the IQVIA™ Longitudinal Prescription (LRx) database [17], which includes information on approximately 80% of all prescriptions reimbursed by Statutory Health Insurance (SHI) funds in Germany. We selected all adult patients receiving a new prescription of vericiguat between 15th of September 2021 and 31st of December 2022. No exclusion criteria have been applied. The initial prescription date was defined as the index date. Further information inferred from prescription records were age, sex, and concomitant medication including GDMT, i.e. the substance classes of beta-blockers (BB), mineralocorticoid receptor antagonists (MRA), renin–angiotensin system inhibitors (RASi; angiotensin converting enzyme inhibitors [ACEi] or angiotensin receptor blockers [ARB] or angiotensin receptor–neprilysin inhibitor [ARNi]), and sodium-glucose co-transporter-2 inhibitors (SGLT2i). Prescriptions provided information including the date of prescription, anatomical therapeutic chemical code, package size, and strength. Drug classes of associated disease areas were used as surrogate for comorbidities [17].

### Titration of vericiguat

Titration patterns of vericiguat treatment were investigated in patients with at least one additional vericiguat prescription not issued on the index date. We assessed the distribution of the first observed dose (“starting dose”) and the maximum dose reached during the respective time period available for analysis. For patients initiated on 2.5 mg vericiguat, the time to up-titration to 5 mg and 10 mg was calculated, respectively.

### Adherence and persistence to treatment

Adherence to vericiguat treatment was investigated in patients with at least one additional vericiguat prescription not yet issued on the index date by calculating the medical possession ratio (MPR) [18]. MPR was defined as the number of days of vericiguat supply divided by the number of days of treatment duration. Treatment duration and days of supply of the last prescription were not included in the definition of the MPR, because this last prescription would have been included as adherence of 100% per definition and would thus have led to an overestimation of the true MPR. In accordance with previous research, adherence to vericiguat treatment was assumed if the MPR exceeded 80% [13, 18, 19].

Persistence of vericiguat treatment during the first 12 months after initiation was operationalized as time until discontinuation and was investigated in patients receiving their first vericiguat prescription until 30th of June 2022 to allow for sufficient follow-up time. A continuous treatment was assumed, if a subsequent prescription occurred either within the days of supply of the previous prescription or during a grace period of 90 days thereafter. Vericiguat prescriptions occurring after the grace period or absence of subsequent prescriptions were considered as discontinuation, with the end date of the last continuous prescription defined as the date of discontinuation. Overlapping days of supply of different packages were assumed to be additive and were thus shifted forward (stockpiling assumption). In sensitivity analyses, varied grace periods of 60 days and 30 days were also investigated. Using a stepwise multivariable Cox proportional hazards regression approach (p > 0.15 for excluding variables), predictors of discontinuation were sought from baseline characteristics of patients starting vericiguat at a dose of 2.5 mg, and hazard ratios (HR) with their 95% confidence intervals (CI) were reported.

### Change of concomitant medication

To analyze changes of concomitant GDMT, information on HF medication was obtained from three-month periods before and after vericiguat initiation. Consequently, only patients with continuously available data during this period were included for this analysis. We calculated the percentage of patients with concomitant HF medication for each drug class separately and in combination.

## Results

### Study population and co-medication at baseline

The study population is characterized in Table 1. In total, 2916 patients received a new prescription of vericiguat between September 2021 and December 2022. Their mean age was 73 ± 13 years (age tertiles: 20–69, 70–81, and ≥ 82 years) and there were 804 (28%) women, 1934 (66%) men, and 178 (6%) patients with unknown sex. The majority of patients received BB (76%) or any RASi (75%) prior to vericiguat initiation. However, only half of the patients were treated with ARNi (53%), MRA (52%) or SGLT2i (51%). Overall, 752 (26%), 745 (26%), and 803 (28%) received two, three or four drug classes of the four foundational therapies, respectively.

**Table 1:** Baseline characteristics of patients initiating vericiguat between September 2021 and December 2022 in Germany

	**Total**	**Women**	**Men**	**Age tertile 1:**	**Age tertile 2:**	**Age tertile 3:**
	**(n = 2916)**	**(n = 804)**	**(n = 1934)**	**20–69 years**	**70–81 years**	** ≥ 82 years**
				**(n = 999)**	**(n = 988)**	**(n = 929)**
Sex						
Female	804 (27.6%)	n/a	n/a	204 (20.4%)	262 (26.5%)	338 (36.4%)
Male	1934 (66.3%)	n/a	n/a	725 (72.6%)	668 (67.6%)	541 (58.2%)
Unknown	178 (6.1%)	n/a	n/a	70 (7.0%)	58 (5.9%)	50 (5.4%)
Age, years	73.0 (12.7)	75.6 (13.3)	72.3 (12.4)	n/a	n/a	n/a
18–50 years	160 (5.5%)	45 (5.6%)	96 (5.0%)	n/a	n/a	n/a
51–60 years	330 (11.3%)	63 (7.8%)	244 (12.6%)	n/a	n/a	n/a
61–70 years	579 (19.9%)	117 (14.6%)	432 (22.3%)	n/a	n/a	n/a
71–80 years	806 (27.6%)	214 (26.6%)	548 (28.3%)	n/a	n/a	n/a
81–90 years	933 (32.0%)	311 (38.7%)	568 (29.4%)	n/a	n/a	n/a
> 90 years	108 (3.7%)	54 (6.7%)	46 (2.4%)	n/a	n/a	n/a
Tertile 1: 20–69 years	999 (34.3%)	204 (25.4%)	725 (37.5%)	n/a	n/a	n/a
Tertile 2: 70–81 years	988 (33.9%)	262 (32.6%)	668 (34.5%)	n/a	n/a	n/a
Tertile 3: ≥ 82 years	929 (31.9%)	338 (42.0%)	541 (28.0%)	n/a	n/a	n/a
HF co-medication^a^						
BB	2207 (75.7%)	614 (76.4%)	1458 (75.4%)	738 (73.9%)	794 (80.4%)	675 (72.7%)
ACEi	468 (16.0%)	153 (19.0%)	285 (14.7%)	119 (11.9%)	161 (16.3%)	188 (20.2%)
ARB	357 (12.2%)	136 (16.9%)	194 (10.0%)	86 (8.6%)	125 (12.7%)	146 (15.7%)
ARNi	1541 (52.8%)	341 (42.4%)	1113 (57.5%)	611 (61.2%)	558 (56.5%)	372 (40.0%)
Any RASi	2174 (74.6%)	572 (71.1%)	1469 (76.0%)	756 (75.7%)	765 (77.4%)	653 (70.3%)
MRA	1519 (52.1%)	360 (44.8%)	1064 (55.0%)	604 (60.5%)	542 (54.9%)	373 (40.2%)
SGLT2i	1479 (50.7%)	328 (40.8%)	1065 (55.1%)	576 (57.7%)	557 (56.4%)	346 (37.2%)
Diuretic	2244 (77.0%)	635 (79.0%)	1487 (76.9%)	684 (68.5%)	786 (79.6%)	774 (83.3%)
Digitalis	290 (9.9%)	98 (12.2%)	182 (9.4%)	75 (7.5%)	107 (10.8%)	108 (11.6%)
Ivabradine	148 (5.1%)	41 (5.1%)	99 (5.1%)	100 (10.0%)	38 (3.8%)	10 (1.1%)
HF drug combinations						
≤ 1 drug class	616 (21.1%)	205 (25.5%)	370 (19.1%)	205 (20.5%)	160 (16.2%)	251 (27.0%)
2 drug classes	752 (25.8%)	235 (29.2%)	480 (24.8%)	184 (18.4%)	251 (25.4%)	317 (34.1%)
3 drug classes	745 (25.5%)	200 (24.9%)	498 (25.7%)	247 (24.7%)	272 (27.5%)	226 (24.3%)
4 drug classes	803 (27.5%)	164 (20.4%)	586 (30.3%)	363 (36.3%)	305 (30.9%)	135 (14.5%)
Non-HF co-medication						
Oral anticoagulant	1661 (57.0%)	456 (56.7%)	1116 (57.7%)	429 (42.9%)	618 (62.6%)	614 (66.1%)
Antiplatelet medication	760 (26.1%)	168 (20.9%)	540 (27.9%)	282 (28.2%)	282 (28.5%)	196 (21.1%)
Lipid-lowering medication	1726 (59.2%)	395 (49.1%)	1226 (63.4%)	563 (56.4%)	661 (66.9%)	502 (54.0%)
Glucose-lowering medication	814 (27.9%)	207 (25.7%)	566 (29.3%)	252 (25.2%)	334 (33.8%)	228 (24.5%)
Anti-depressants	365 (12.5%)	137 (17.0%)	206 (10.7%)	121 (12.1%)	122 (12.3%)	122 (13.1%)
NSAIDs	329 (11.3%)	78 (9.7%)	232 (12.0%)	124 (12.4%)	104 (10.5%)	101 (10.9%)
Antiobstructive medication	682 (23.4%)	164 (20.4%)	481 (24.9%)	215 (21.5%)	268 (27.1%)	199 (21.4%)
Gout medication	791 (27.1%)	149 (18.5%)	597 (30.9%)	220 (22.0%)	306 (31.0%)	265 (28.5%)

Data are n (%) or mean (SD)

*HF* heart failure, *BB* beta-blockers, *MRA* mineralocorticoid receptor antagonists, *RASi* renin–angiotensin system inhibitors, *ACEi* angiotensin converting enzyme inhibitors, *ARB* angiotensin receptor blockers, *ARNi* angiotensin receptor–neprilysin inhibitor [ARNi]), *SGLT2i* sodium-glucose co-transporter-2 inhibitors, *NSAIDs* non-steroidal anti-inflammatory drug, *n/a* not available

^a^Use of co-medication was assessed during the three months prior to vericiguat initiation

Women started on vericiguat were older than men (76 ± 13 vs. 72 ± 12 years). While there were no differences regarding the use of BB, women received ARNi (42% vs. 58%), MRA (45% vs. 55%) and SGLT2i (41% vs. 55%) less often than men. Accordingly, the percentage of patients receiving all four pillars of HF drug therapy was lower in women compared to men (20% vs. 30%).

The percentage of women increased with age, and was 20% in the youngest and 36% in the oldest age category, respectively. When compared to younger patients between 20 to 69 years, individuals aged at least 82 years received ARNi (61% vs. 40%), MRA (60% vs. 40%) and SGLT2i (58% vs. 37%) less often. Consistently, younger patients were treated more frequently with quadruple therapy (36% vs. 15%).

### Titration patterns in patients initiating vericiguat

Among 2916 patients, 2129 (73%) had at least one additional vericiguat prescription that was not issued on the index date and, therefore, were eligible for this analysis. Of those, mean age was 73 ± 13 years, and there were 565 (27%) women, 1423 (67%) men and 141 (7%) patients with unknown sex. The median follow-up time was 150 (quartiles 73, 253) days. Overall, as depicted in Table 2, the share of patients for the first observed dose (“starting dose”) of vericiguat was 84% for 2.5 mg, 12% for 5 mg and 4% for 10 mg. About one third each remained on the 2.5 mg or 5 mg as their maximum dose, while 36% were up-titrated to the full dose of 10 mg. The median time for up-titration from 2.5 mg to 5 mg was 17 (11, 33) days, and the respective time from 2.5 mg to 10 mg 37 (25, 64) days. There were no sex- or age-specific differences regarding the starting dose of vericiguat or up-titration time. However, women and patients in the highest age category reached the maximum dose of 10 mg less often, when compared to men (34% vs. 37%) or to younger patients (20–69 years: 38%, 70–81 years: 37%, > 82 years: 33%).

**Table 2:** Up-titration patterns in patients initiating vericiguat

	**Total** **(n = 2129)**	**Women** **(n = 565)**	**Men** **(n = 1423)**	**Age tertile 1:** **20–69 years** **(n = 716)**	**Age tertile 2:** **70–81 years** **(n = 732)**	**Age tertile 3:** ** ≥ 82 years** **(n = 681)**
First observed dose (“starting dose”)						
2.5 mg	1792 (84.2%)	477 (84.4%)	1198 (84.2%)	603 (84.2%)	615 (84.0%)	574 (84.3%)
5 mg	254 (11.9%)	68 (12.0%)	167 (11.7%)	86 (12.0%)	92 (12.6%)	76 (11.2%)
10 mg	83 (3.9%)	20 (3.5%)	58 (4.1%)	27 (3.8%)	25 (3.4%)	31 (4.6%)
Maximal dose reached						
2.5 mg	652 (30.6%)	158 (28.0%)	444 (31.2%)	201 (28.1%)	230 (31.4%)	221 (32.5%)
5 mg	708 (33.3%)	213 (37.7%)	448 (31.5%)	245 (34.2%)	228 (31.1%)	235 (34.5%)
10 mg	769 (36.1%)	194 (34.3%)	531 (37.3%)	270 (37.7%)	274 (37.4%)	225 (33.0%)
Time (days) until up-titration						
to 5 mg	17.0 (11.0–33.0)	16.0 (9.0–34.0)	17.0 (11.0–32.0)	20.0 (13.0–45.0)	16.5 (11.0–29.0)	14.0 (8.0–30.0)
to 10 mg	37.0 (25.0–64.0)	37.0 (24.5–64.5)	39.0 (26.0–64.0)	41.0 (26.0–73.0)	36.5 (25.0–59.0)	34.0 (24.0–55.0)

Data are n (%) or median (quartiles)

### Adherence and persistence to vericiguat

**Fig. 1:**
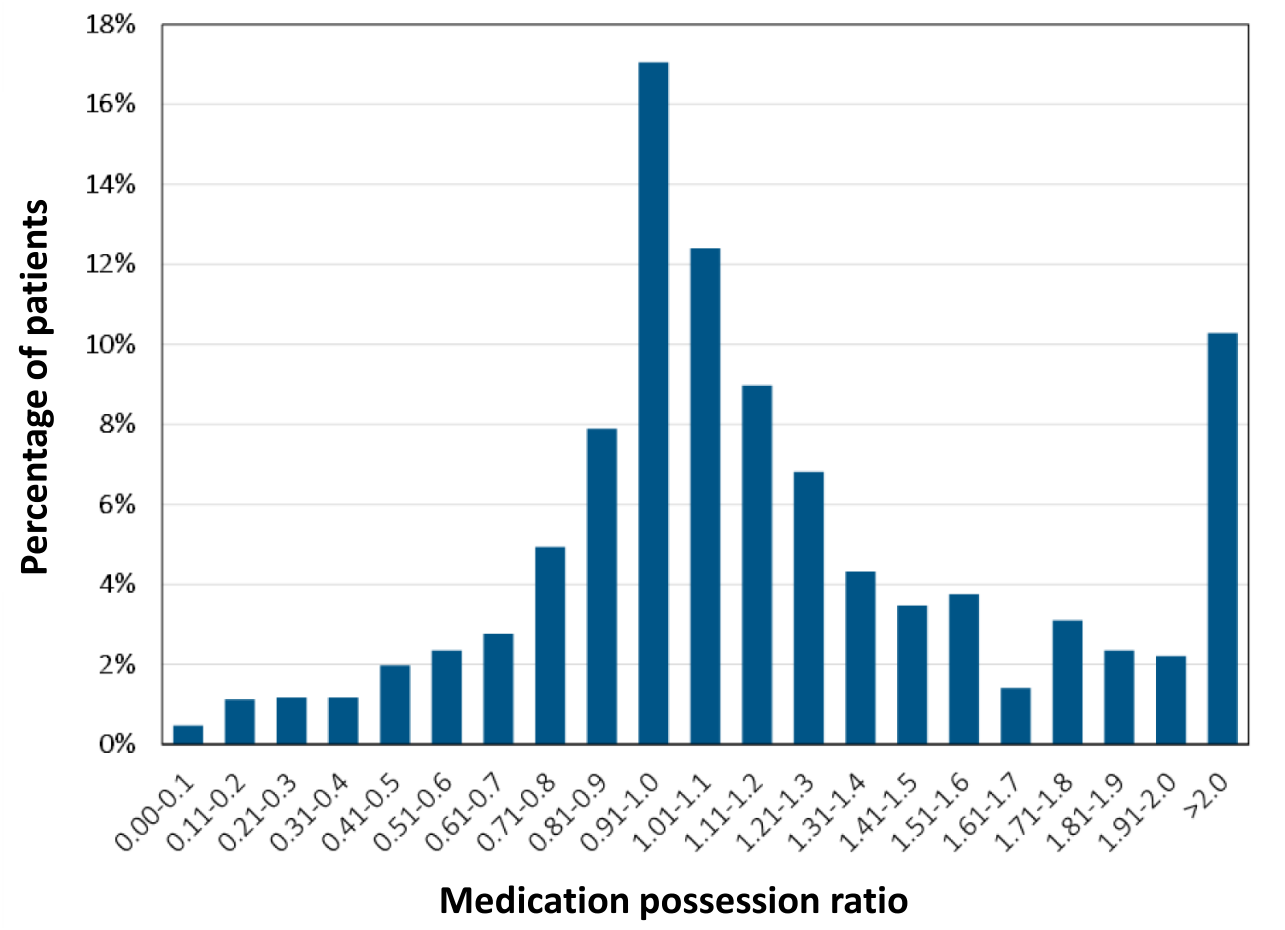
Adherence to treatment with vericiguat. Medication possession ratio (MPR) was defined as ratio of number of days a patient is stocked with medication by number of days per treatment period. MPR ≥ 0.80 indicates adherent use of vericiguat. MPR > 1.0 points towards stockpiling

Adherence to vericiguat treatment was investigated in 2129 eligible patients and is depicted in Fig. 1. In the majority of patients (87%), adherence to vericiguat was high (MPR ≥ 80%). We found no sex- or age-specific differences: the proportion of patients with high adherence to vericiguat was similar in women (88%) and men (86%), as well as across age categories (20–69 years: 84%, 70–81 years: 88%, ≥ 82 years: 88%).

**Fig. 2:**
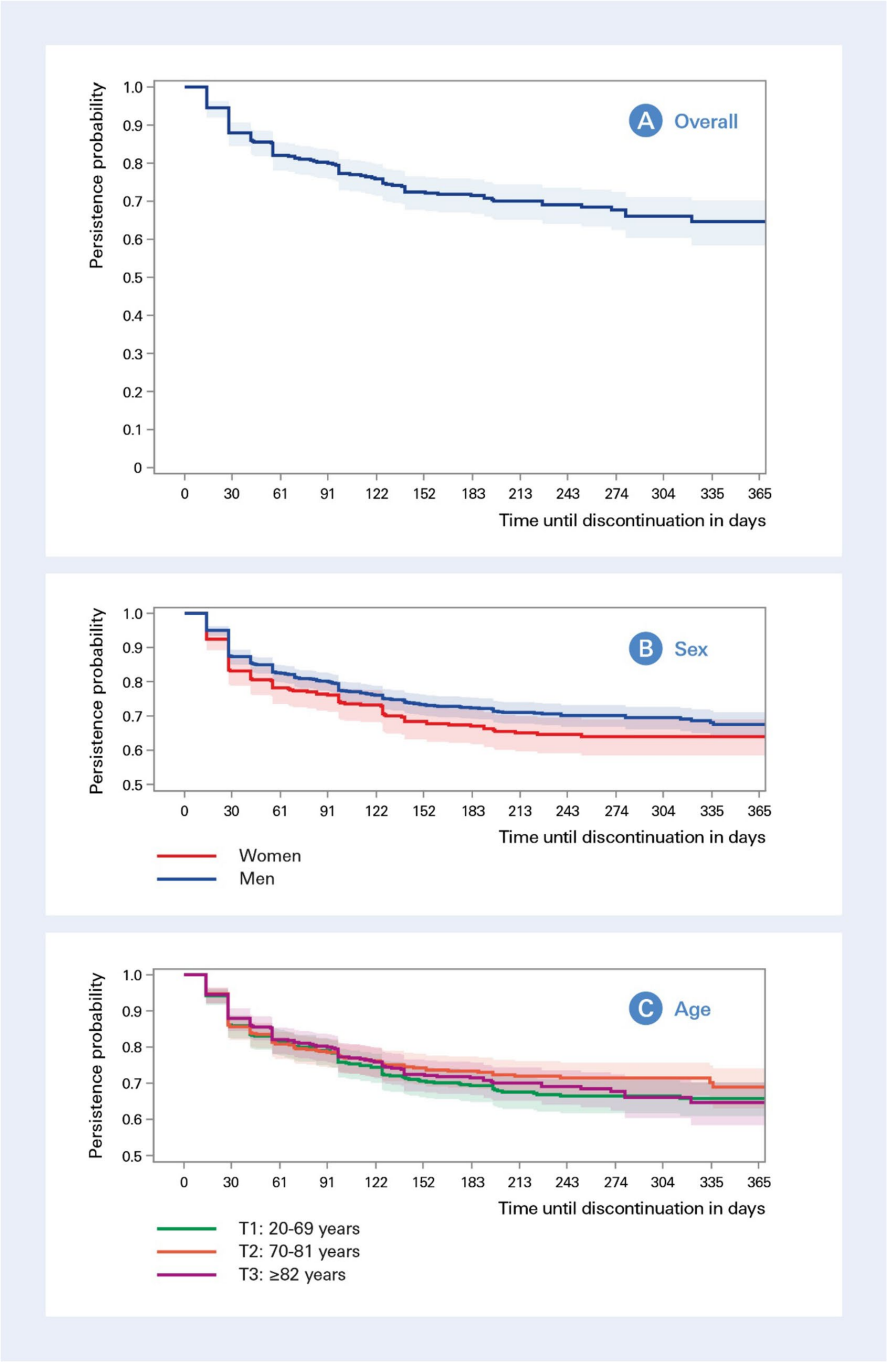
Persistence to treatment with vericiguat. The Kaplan-Meier plots show the probability of persistently using vericiguat over a 12-month period in 1402 patients initiating vericiguat between September 2021 and June 2022 for all patients (**A**), stratified by sex (**B**), stratified by age tertiles (**C**)

Among the 1402 patients eligible for persistence analysis, the majority (67%) persistently used vericiguat in the first 12 months after initiation when allowing for a therapy gap of 90 days (Fig. 2A). Overall, discontinuation predominantly occurred within the first two months (i.e. in 18%), and was observed less often afterwards (15%). The median time until discontinuation was 42 (28–98) days. In sensitivity analyses allowing for 60-day or 30-day grace periods, the proportion of patients with uninterrupted use were 62% and 52%, respectively (Figs. 3 and 4 in the Supplementary file). As illustrated in Fig. 2B, there was a trend for higher discontinuation in women than in men, yet with overlapping confidence intervals and differences diminished towards the end of observation period. Nevertheless, the median time until discontinuation was shorter in women when compared to men (29 vs 42 days). No differences for persistence to vericiguat regarding age became apparent (Fig. 2C). In multivariable Cox regression considering variables from Table 1, the use of BB, new oral anticoagulants, and lipid-lowering medication were associated with a lower risk of discontinuation, while non-steroidal anti-inflammatory drugs (NSAIDs) and gout medication increased the probability of discontinuation (Table 4 in the Supplementary file).

### Increase of concomitant GDMT after initiation with vericiguat

The co-medications three months before and after initiation with vericiguat of 1416 eligible patients are detailed in Table 3. The number of patients receiving HF-specific medication increased across all four pillars of HF therapy, and to a lesser extent for medication unrelated to HF, e.g. oral anticoagulants (58% vs. 64%). The strongest increase for GDMT was observed for SGLT2i from 51 to 73%. The number of patients receiving all four pillars of foundational therapy increased from 408 (29%) to 619 (44%).

**Table 3:** Change of co-prescribed medication during the three months before and after initiation with vericiguat

	**Prior to vericiguat initiation** **(n = 1416)**	**After to vericiguat initiation** **(n = 1416)**
HF co-medication		
BB	1113 (78.6%)	1202 (84.9%)
ACEi	236 (16.7%)	204 (14.4%)
ARB	179 (12.6%)	168 (11.9%)
ARNi	801 (56.6%)	949 (67.0%)
Any RASi	1113 (78.6%)	1193 (84.3%)
MRA	769 (54.3%)	943 (66.6%)
SGLT2i	724 (51.1%)	1040 (73.4%)
Diuretic medication	1124 (79.4%)	1243 (87.8%)
Digitalis	141 (10.0%)	175 (12.4%)
Ivabradine	82 (5.8%)	104 (7.3%)
HF drug combinations		
≤ 1 drug class	252 (17.8%)	103 (7.3%)
2 drug classes	381 (26.9%)	319 (22.5%)
3 drug classes	375 (26.5%)	375 (26.5%)
4 drug classes	408 (28.8%)	619 (43.7%)
Non-HF co-medication		
Oral anticoagulant	818 (57.8%)	910 (64.3%)
Antiplatelet medication	382 (27.0%)	418 (29.5%)
Lipid-lowering medication	881 (62.2%)	960 (67.8%)
Glucose-lowering medication	445 (31.4%)	431 (30.4%)
Anti-depressant	171 (12.1%)	200 (14.1%)
NSAIDs	177 (12.5%)	157 (11.1%)
Antiobstructive medication	346 (24.4%)	349 (24.6%)
Gout medication	414 (29.2%)	436 (30.8%)

*HF* heart failure, *BB* beta-blockers, *MRA* mineralocorticoid receptor antagonists, *RASi* renin–angiotensin system inhibitors, *ACEi* angiotensin converting enzyme inhibitors, *ARB* angiotensin receptor blockers, *ARNi* angiotensin receptor–neprilysin inhibitor [ARNi]), *SGLT2i* sodium-glucose co-transporter-2 inhibitors, *NSAIDs* non-steroidal anti-inflammatory drugs

## Discussion

Our study has three major findings. First, patients in this real-world HF cohort were slightly more often women and were older than reported in the landmark clinical trial [8]. Second, the up-titration time was in line with recommendations. Yet, while approximately 70% reached the 5 mg or 10 mg dose, only 36% were up-titrated to 10 mg, with women and older patients reaching the maximum dose of 10 mg less often and also receiving other GDMT less frequently. However, the proportion of patients receiving all four pillars of GDMT increased from 29% before vericiguat initiation to 44% afterwards. Third, adherence and persistence to vericiguat appeared to be satisfactory, which was consistent across age categories.

### Baseline characteristics

The here studied real-world population differed from patients investigated in the VICTORIA trial [8]. In our study, the proportion of women was slightly higher (28% vs. 24%) and patients were approximately six years older (mean age 73 vs. 67 years). These findings compare well with prior evidence on discordance between clinical characteristics of trial participants and the HF population at large [20]. Moreover, our results are consistent with a report of Nguyen et al., who investigated eligibility for vericiguat among a real-world HF population in Sweden: when applying guideline and product label criteria, the mean age of eligible patients was 78 years and 28% were women [21].

Regarding the concomitant use of GDMT, 60% of patients received three drug classes (BB, MRA, and any RASi) and 15% received ARNi in the VICTORIA trial. In our study, the amount of patients treated with at least three drug classes (BB, MRA, any RASI, or SGLT2i) was lower (53%), whereas the proportion of patients receiving ARNi was higher (53%). Comparability is limited though, since ARNi had been introduced to the market approximately one year before the recruitment of VICTORIA started, and SGLT2i were not yet part of the foundational HF therapy at that time.

### Titration patterns of vericiguat

The titration regimen of vericiguat consists of 2 up-titration visits each 2 weeks apart to reach the maximal dose of 10 mg, resulting in a total titration time of 28 days from 2.5 mg to 10 mg. In our study, the median times until up-titration were 17 days for the first step (2.5 mg to 5 mg), and 37 days for the total period (2.5 mg to 10 mg). Considering the many challenges of real-world HF care including regular blood pressure monitoring, the potential need for short-term follow-up visits, and interdisciplinary cooperation between general practitioners and cardiologists, these findings are encouraging and demonstrate the feasibility of the up-titration schedule. Regarding dosages, approximately 70% of the patients were up-titrated to the 5 mg or 10 mg dose, but only 36% reached the target dose of 10 mg. Furthermore, women and patients in the high-age tertile were less often up-titrated to the maximal dose when compared to men and younger patients. These results corroborate the findings of previous studies reporting on sex- and aged-based disparities for other GDMT [10, 22–25], and emphasize the need for enhanced attention to these subgroups in order to reduce inequity in GDMT. For instance, the EVOLUTION-HF study investigated the HF drug therapy including titrations patterns in 266,589 patients from Japan, Sweden, and the US [24]. In this study, achievement of target doses was lower for women than for men (e.g., only 24% of women reached the target dose of ARNi compared to 30% of men). Similar yet smaller differences were observed when comparing patients aged ≥ 70 years vs. < 70 years.

### Adherence and persistence to vericiguat

An important finding of this study was that adherence to treatment with vericiguat was fairly high. In the VICTORIA trial, adherence to vericiguat use was greater than 80% in 94% of the patients in the vericiguat group, while it was observed in 87% of the patients in our real-world setting. Of note, adherence was consistently high for both sexes and all age categories. Considering the higher age of our population and the well-known challenges for GDMT implementation, these discrepancies between clinical trial and real-world appear encouragingly small. No study has investigated adherence to vericiguat so far, but there are reports on adherence to other GDMT in HF. In a nation-wide cohort study in Norway including 54,899 patients between 2014 and 2020, the proportion of patients with high drug adherence were 83% for BB, 81% for any RASi, and 61% for MRA [26]. Interestingly, adherence to ARNi alone was highest (84%), whereas high adherence to dual or triple HF therapy was observed only in 42% and 5%, respectively. Similar results for ARNi have been reported by other real-world studies from the US [27], and Germany [28]. Hence, adherence to vericiguat in our study seems to accord well with previously reported adherence to other novel GDMT (i.e. ARNi).

In the present study, 67% of the patients persistently used vericiguat within the first 12 months after initiation, which seems acceptable in comparison to previous findings for other GDMT. In the study of Wachter et al., persistence to ARNi after one year was 71%. In the EVOLUTION-HF study, respective discontinuation rates in the first year after initiation for SGLT2i (dapagliflozin), ARNi, ACEi, ARB, BB, and MRA were 23%, 26%, 38%, 33%, 25%, and 42%, respectively [24]. However, this comparison is flawed, since grace periods were not reported. Ødegaard et al. applied a 30-day grace period and found that the 1-year persistence to BB, RASi, and MRA was 72%, 71%, and 48%, respectively, which compares to 52% persistence to vericiguat found in our 30-day sensitivity analysis. Similar to our findings, discontinuation mainly occurred in the first months after initiation, which might be indicative for a vulnerable phase for drug discontinuation. In our analysis, we found no differences regarding age, but a trend for slightly higher discontinuation of vericiguat in women than in men. High persistence across age categories contrasts with findings from previous studies on GDMT, whereas higher discontinuation rates for women have already been reported [24, 28]. Nevertheless, our results might point towards a good safety profile of vericiguat in real-world settings. In our analysis, NSAIDs and gout medication were found to be independent predictors of vericiguat discontinuation. Reasons for non-adherence and drivers of discontinuation warrant further investigation.

### Therapeutic intensification of other GDMT following initiation of vericiguat

The current study found that the initiation of vericiguat was associated with an increased proportion of patients on quadruple therapy overall and for each substance class separately. Such mutual enhancement has not been observed so far in similar studies addressing GDMT in HF. For instance, Bhatt et al. reported that other disease-modifying medication declined in the three months after the initiation of ARNi: the proportion of patients receiving BB and MRA decreased from 92 to 83% and from 39 to 32%, respectively [27]. Similarly, in the study of Wachter et al., the use of BB and MRA dropped in the subsequent six months after initiating ARNi by general practitioners (BB: from 89 to 84%, MRA: from 61 to 54%) or cardiologists (BB: from 92 to 86%, MRA: from 72 to 63%). The discordant findings in our study might have several reasons. First, the clinical characteristics from this study likely differed since the initiation of vericiguat requires a recent WHF event. Second, it is conceivable that this WHF event might have prompted physicians initiating vericiguat to take a particularly critical look at and optimize HF therapy. This might be also reflected by the concomitant surge of use of ARNi during the period of vericiguat implementation. Third, the increased use of SGLT2i after initiating vericiguat might be explained largely by its recent market entry in 2021. Fourth, vericiguat is targeting a distinct biological pathway in comparison to other HF medications [29]. Consequently, discontinuation due to increased serum potassium levels or markedly lowered blood pressure levels seems less likely. In a post-hoc analysis of the VICTORIA trial, the mean systolic blood pressure showed a small decline after initiation in both treatment arms and returned to baseline afterwards [30]. Effects were similar for the whole study sample as well as for vulnerable subgroups (i.e., patients older than 75 years, with baseline systolic blood pressure < 100 mmHg, or pre-treatment with ARNi). Finally, the intensification of GDMT in the here reported population might have been facilitated by worse baseline levels than in the other two studies mentioned above.

Taken together, the concern that the addition of a further substance class could be at the expense of other GDMT appears unfounded in connection with vericiguat. On the contrary, initiation of vericiguat appears to facilitate therapeutic intensification and GDMT optimization. More studies are needed to validate these findings.

### Strengths and limitations

Several strengths and limitations of the current study need to be considered. It is the first analysis of real-world use of vericiguat on a nation-wide level and, thus, may contribute to a better understanding of novel GDMT implementation in routine clinical practice. Administrative data such as pharmacy claims data provide an effective and reliable way to assess treatment patterns, adherence and persistence [18]. However, the absence of clinical data limits interpretation and comparability with prior findings. Furthermore, generalizability is reduced by the fact that only prescriptions from office-based pharmacies were available from the database. Consequently, initial prescriptions of vericiguat during hospitalization might have been missed potentially leading to an overestimation of patients initiating vericiguat on 5 mg/10 mg.

## Conclusions

Although in this prescription claims analysis the age of patients was higher than in clinical trials studying vericiguat, adherence and persistence appeared satisfactory across age categories. However, women and older patients were less often up-titrated to the target dose of vericiguat and received other GDMT less frequently. The initiation of vericiguat was associated with therapeutic intensification for other GDMT. Drivers and barriers to vericiguat up-titration and implementation of other GDMT, in particular in women and elderly, need to be investigated further.

### Supplementary Information

Supplementary file 1 (PDF 420 kb)

Supplementary file 2 (PDF 420 kb)

Supplementary file 3 (DOCX 15.1 kb)

## Data Availability

This study is based on a proprietary database from IQVIA Commercial GmbH & Co. OHG. Therefore, access to data is restricted, it cannot be accessed publicly.
