# Supplementary figures and images for: Real-world characteristics and use patterns of patients treated with vericiguat: A nationwide longitudinal cohort study in Germany
Source: Eur J Clin Pharmacol. 2024 Mar 12;80(6):931–40. doi: 10.1007/s00228-024-03654-0 (PMC11098883; doi:10.1007/s00228-024-03654-0)

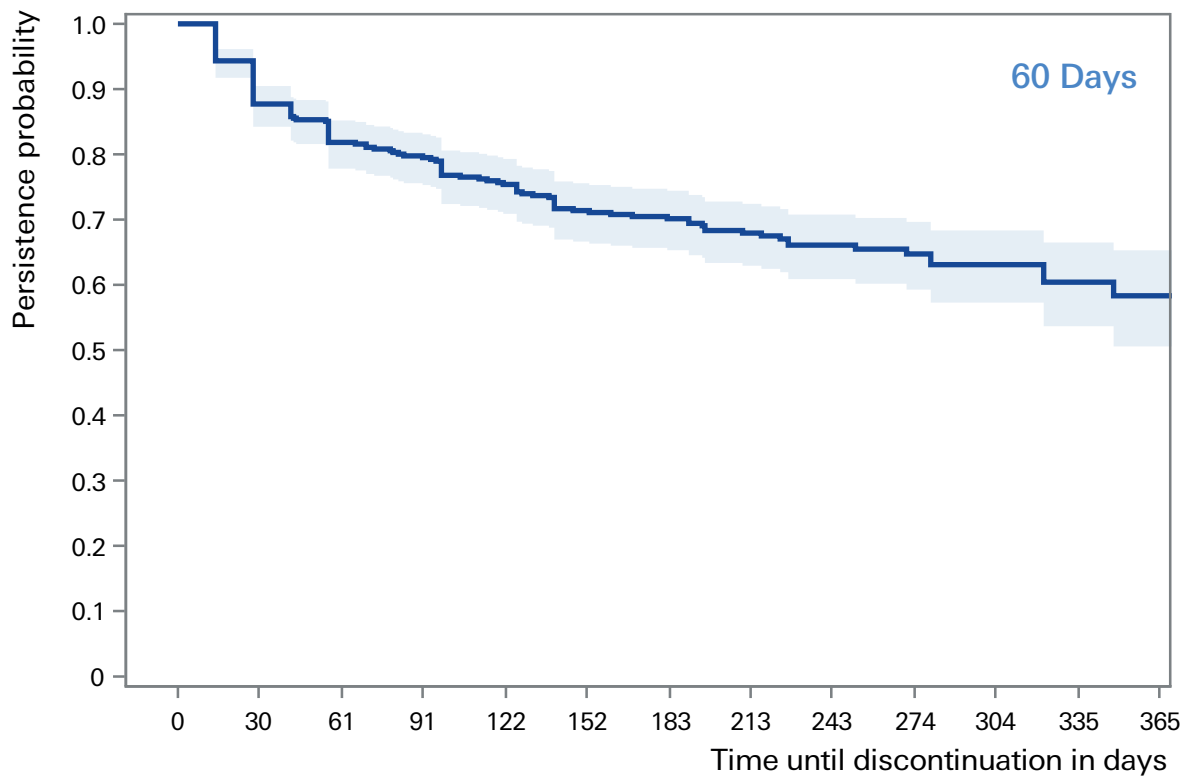

Supplement: Supplementary file 1 — (PDF 420 kb) [file 228_2024_3654_MOESM1_ESM.pdf]

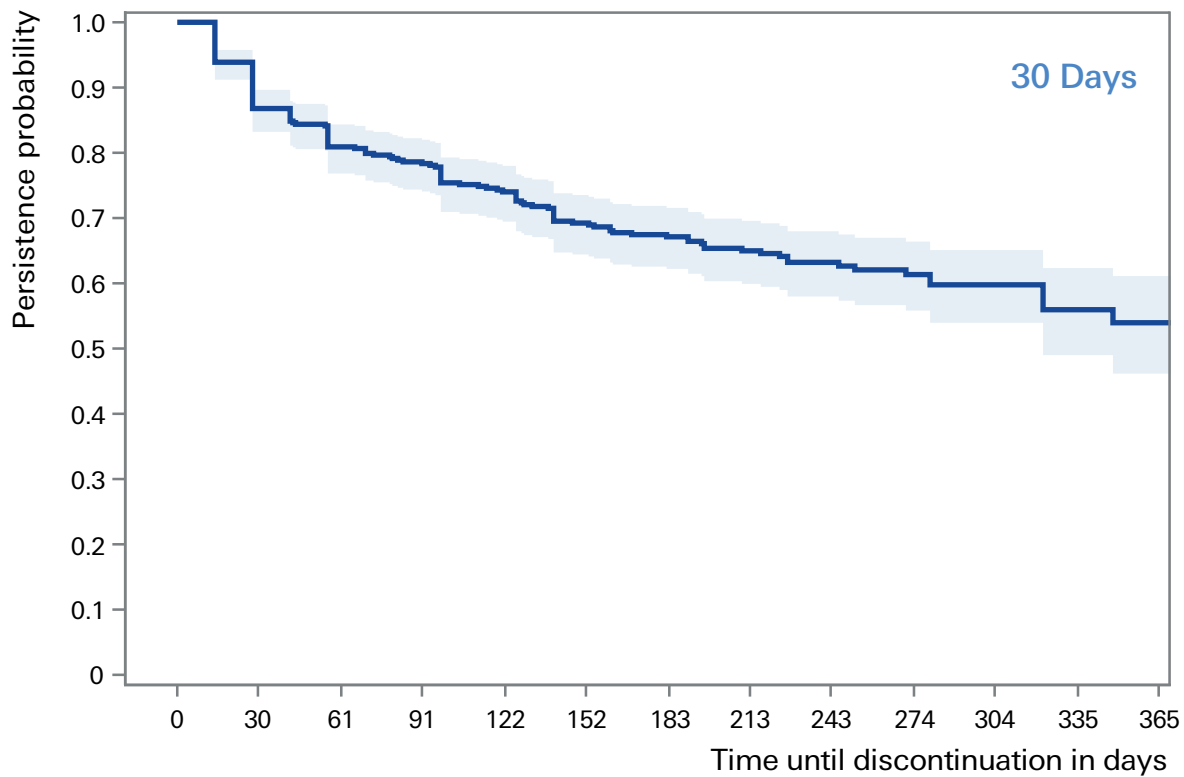

Supplement: Supplementary file 2 — (PDF 420 kb) [file 228_2024_3654_MOESM2_ESM.pdf]
