# Supplementary material for: Real-world characteristics and use patterns of patients treated with vericiguat: A nationwide longitudinal cohort study in Germany
Source: Eur J Clin Pharmacol. 2024 Mar 12;80(6):931–40. doi: 10.1007/s00228-024-03654-0 (PMC11098883; doi:10.1007/s00228-024-03654-0)
Supplement: Supplementary file 3 — (DOCX 15.1 kb) [file 228_2024_3654_MOESM3_ESM.docx]

Table 4: Predictors of treatment discontinuation in 821 patients starting vericiguat at a dose of 2.5 mg

| **Variables** | **Categories** | **Number of all patients** | **Number of patients with discontinuation** | **Hazard ratio  (95% CI)** | **p-value** |
| --- | --- | --- | --- | --- | --- |
| Beta-blockers | No | 157 | 52 | Ref. | 0.005 |
|  | Yes | 664 | 133 | 0.62 (0.45–0.87) |  |
| New oral anticoagulants | No | 412 | 105 | Ref. | 0.099 |
|  | Yes | 409 | 80 | 0.78 (0.58–1.05) |  |
| Lipid-lowering medication | No | 287 | 84 | Ref. | 0.004 |
|  | Yes | 534 | 101 | 0.64 (0.48–0.87) |  |
| NSAIDs | No | 716 | 151 | Ref. | 0.026 |
|  | Yes | 105 | 34 | 1.53 (1.05–2.23) |  |
| Gout medication | No | 570 | 123 | Ref. | 0.073 |
|  | Yes | 251 | 62 | 1.33 (0.97–1.82) |  |

Ref. = referent. The table shows the results of multivariable modelling using all baseline characteristics from Table 1 (p > 0.15 for exclusion from the model).

NSAIDs = nonsteroidal anti-inflammatory drugs
